# LINC00963/miR-4458 regulates the effect of oxaliplatin in gastric cancer by mediating autophagic flux through targeting of ATG16L1

**DOI:** 10.1038/s41598-021-98728-9

**Published:** 2021-10-25

**Authors:** Meng Hou, Chao Li, Shunbin Dong

**Affiliations:** 1 Department of Obstetrics and Gynecology, The First Affiliated Hospital of Xi’an Jiaotong University, Xi’an, 710061 China; 2 Department of General Surgery, The First Affiliated Hospital of Xi’an Jiaotong University, Xi’an, 710061 Shaanxi China; 3 Department of Hepatobiliary Surgery, The First Affiliated Hospital of Xi’an Jiaotong University, Xi’an, 710061 Shaanxi China

**Keywords:** Cancer therapy, Gastrointestinal cancer

## Abstract

Oxaliplatin resistance is the greatest obstacle to the management of local recurrence in gastric cancer patients after surgery. Accumulating evidence has suggested that inhibiting autophagy may be a novel approach for reversing resistance to oxaliplatin treatment. In this manuscript, we aimed to investigate the role of LINC00963 in regulating autophagy and oxaliplatin resistance. qRT-PCR, immunochemistry staining, and western blotting were used to detect gene expression. Plasmids were used to up- and downregulate the expression of LINC00963 and miR-4458. A caspase 3/7 activity kit and flow cytometry were used to detect the apoptosis rate. CCK8 and Transwell assays were used to test cell proliferation and migration, respectively. Transmission electron microscopy and a dual fluorescent lentivirus autophagy system were used to evaluate autophagic flux. Dual luciferase reporter gene assays and RNA pulldown assays were used to evaluate the potential crosstalk. LINC00963 was highly expressed in gastric cancer patients and cell lines. In addition, high LINC00963 expression was found to be associated with poor prognosis and local recurrence in gastric cancer patients, indicating that LINC00963 might be involved in oxaliplatin resistance. Moreover, we found that LINC00963 was aberrantly highly expressed in oxaliplatin-resistant SGC-7901 (SGC-7901-R) cells and promoted proliferation and migration and reduced the apoptosis rate in SGC-7901-R cells. Furthermore, among all potential target microRNAs, miR-4458 was found to be negatively regulated by LINC00963 both in vivo and in vitro. In addition, miR-4458 overexpression led to impaired proliferation and migration and enhanced cell apoptosis and G1 arrest in SGC-7901-R cells. Further RNA pulldown and dual luciferase reporter gene assays indicated the interaction between LINC00963 and miR-4458. Moreover, we found enhanced autophagic flux in SGC-7901-R cells compared with SGC-7901 cells; in addition, an inhibitor of autophagy induced apoptosis in SGC-7901-R cells. Then, we found that downregulation of LINC00963 expression and upregulation of miR-4458 expression significantly suppressed autophagic flux in SGC-7901-R cells. Based on starBase V3.0 and dual luciferase reporter gene assays, we predicted and confirmed that ATG16L1 might be the target of miR-4458 to regulate autophagy. In conclusion, LINC00963 and miR-4458 are potential biomarkers for predicting the overall survival of gastric cancer patients. Moreover, targeting LINC00963 to inhibit autophagic flux sensitizes gastric cancer cells to oxaliplatin treatment, suggesting that it is a potential novel therapeutic target for improving oxaliplatin sensitivity.

## Introduction

Gastric cancer is characterized by aggressiveness and a propensity for local recurrence^1^. Currently, oxaliplatin-based chemotherapy, such as XELOX, is the mainstay treatment for controlling local recurrence of gastric cancer after surgery^2,3^. However, oxaliplatin resistance always impairs the efficacy of oxaliplatin-based chemotherapy, leading to local recurrence in gastric cancer patients^3^.

In the recent decade, numerous studies have emphasized the importance of autophagy in mediating oxaliplatin sensitivity^4–6^. For example, oxaliplatin has been shown to induce autophagic flux in various cancers, indicating the potential crosstalk between oxaliplatin and autophagy^7–9^. Additionally, enhanced autophagic flux in oxaliplatin-resistant cancer cells suggests that autophagy plays an important role in inducing oxaliplatin resistance^9,10^. In fact, inhibition of autophagic flux in hepatocellular carcinoma through downregulation of ATG7 expression or chloroquine treatment was found to successfully induce apoptosis and reverse oxaliplatin resistance^11^. In addition, Siyuan et al.^12^ demonstrated that downregulating PFKFB3 expression impaired oxaliplatin-induced autophagic flux, thereby increasing oxaliplatin cytotoxicity. Moreover, inhibition of autophagy might be necessary to improve the efficacy of oxaliplatin. For instance, Yanli et al. reported that the enhanced effect of oxaliplatin following WASF knockdown was dependent on the inhibition of ATG12-mediated autophagy^8^. The ATG5-ATG12-ATG16L1 complex is an important pathway for promoting autophagic flux. Here, ATG16L1 plays an important role in stabilizing the ATG5-ATG12 complex^13^. In this study, we adopted starBase V3.0 for the prediction of potential microRNAs that potentially target ATG16L1’s 3' UTR. Among them, miR-4458, a miRNA that not only targets ATG16L1 but also serves as a downstream target of LINC00963, was identified. Previous studies have shown that miR-4458 is a tumour suppressor in oesophageal squamous cell carcinoma, breast cancer, and hepatocellular carcinoma, among others^14–16^. However, its specific role in gastric cancer remains unclear.

Accumulating evidence has indicated the important role of long noncoding RNAs in regulating drug resistance^17^. For example, LINC00963 was found to play a key role in promoting multidrug resistance in head and neck carcinoma ^18^. In addition, LINC00963 knockdown resulted in impaired migration, invasion and colony formation in head and neck carcinomas, as well as decreased levels of ABCG2 and ABCB5, key regulators of multidrug resistance^19^. ABCB5 is one of the ATP-binding cassette (ABC) transporters that negatively regulates 5-fluorouracil and platinum resistance^20^, whereas ABCG2 was found to regulate oxaliplatin resistance in colorectal cancer cells^21^. Consequently, we assumed that LINC00963 might be involved in promoting oxaliplatin resistance.

In short, we hypothesized that LINC00963 might be involved in promoting oxaliplatin resistance in gastric cancer by inducing autophagy through targeting of ATG16L1; further online bioinformatics tools predicted that LINC00963 regulated ATG16L1 expression by sponging miR-4458. Therefore, we sought to unravel LINC00963’s role in mediating autophagy and oxaliplatin sensitivity in gastric cancer. In this manuscript, we aimed to further elucidate the specific role and molecular mechanism of autophagy in mediating oxaliplatin resistance in gastric cancer.

## Methods

### Patient recruitment

We recruited 289 gastric cancer patients from 2011 to 2015 at the First Affiliated Hospital of Xi`an Jiaotong University. We collected gastric cancer and adjacent normal gastric tissues during surgery without influencing the standard pathological examination. All recruited patients were treated with XELOX (capecitabine and oxaliplatin) after surgery. Follow-ups were carried out every 3 months, during the first year following surgery, every 6 months in the second year, and every year during the last 3 years. During the follow-up, patients were physically examined, and chest CT, routine blood tests and cranial CT were also performed where applicable. In cases where local recurrence was observed, the affected patients were categorized as oxaliplatin-resistant and subjected to additional treatment.

### Statements for experiments that involved human beings

The study was explained to patients in advance, with all of them signing informed consent prior to recruitment. The study met the guidelines described by the Declaration of Helsinki and was approved by the ethics committee of the First Affiliated Hospital of Xi`an Jiaotong University (2019 G-50).

### Cell cultures and transfections

SGC-7901, MKN45 and MKN74 cell lines were purchased from the American Type Culture Collection (ATCC, Manassas, VA, USA) and then cultured in RPMI 1640 medium supplemented with 10% foetal bovine serum (FBS) (Gibco, USA). All cultures were maintained at 37℃ and 5% CO_2_. We generated constructs for LINC00963 and miR-4458 knockdown, as well as miR-4458 overexpression, using the GenePharm kit (Shanghai, China) and then transfected them into target cells according to the manufacturer’s instructions for the Lipofectamine 3000 kit (Invitrogen, Shanghai, China).

### RNA extraction and quantitative real-time polymerase chain reaction (qRT-PCR)

Total RNA was extracted from the patients’ gastric cancer tissues and adjacent normal tissues as well as gastric cell lines using TRIzol reagent (Invitrogen, Shanghai, China). The RNA was reverse-transcribed using the ReverTra Ace kit (Toyobo Co., Ltd., Osaka, Japan) according to the manufacturer’s instructions, and then, qRT-PCR targeting LINC00963 and miR-4458 was carried out using Thunderbird SYBR qPCR Mix (Toyobo Co., Ltd., Osaka, Japan) in a LightCycler 2.0 system (Roche Molecular Systems, Inc., Pleasanton, CA, USA). GAPDH and U6 were also included as controls for LINC00963 and miR-4458, respectively.

### RNA pulldown

We purchased biotinylated miR-4458 probes from Genecreate (Wuhan, China). Specifically, miR-4458-Wt was used as the wild-type probe, whereas miR-4458-Mut had a mutated binding site between miR-4458 and LINC00963. Bonded RNA was harvested using M-280 Streptavidin-coated MagneSphere particles, followed by elution and purification. Finally, qRT-PCR was used to reveal the levels of LINC00963 expression.

### Dual luciferase reporter gene assay

We constructed pcDNA 3.1 plasmids comprising wild-type or mutated LINC00963 (the miR-4458 binding region was mutated) promoter regions using the pmirGLO luciferase vector (Promega, MA, USA). Then, we transfected the SGC-7901 cell line with LINC00963-WT or LINC00963-Mut with miR-4458 or NC mimics. Thereafter, we analysed luciferase activity in the transfected cells using the Dual-Luciferase Reporter Assay System (Promega, MA, USA) according to the manufacturer’s instructions.

### Analysis of cell proliferation

We analysed cell viability using the Cell Counting Kit-8 assays. Briefly, we seeded 1 × 10^3^ cells in each well of a 96-well plate and then cultured them for 24, 48 and 72 h. We added 10 µL of CCK‑8 to each cell sample, incubated the samples for 2 h, and then measured the resulting absorbance at 450 nm.

### Analysis of apoptosis

We investigated the rate of apoptosis in vitro using the FAM-FLICA Caspase 3/7 assay kit. In summary, we seeded 1 × 10^3^ cells in each well of a 96-well plate and then added FLICA to each well, with a 60-min incubation. Finally, we detected Caspase 3/7 activity by fluorescence microscopy. The FITC Annexin V Apoptosis Detection Kit I (BD Pharmingen TM, New Jersey, USA) was used for apoptosis testing. Target cells were stained with Annexin V–FITC at room temperature for 15 min, followed by flow cytometry to detect the fluorescence intensity.

### Analysis of autophagy

Cells were transfected with the SensGFP-StubRFP-LC3 virus, which was constructed by GeneChem. After 24 h of transfection, cells were selected with puromycin. Cells (1 × 10^4^) were plated into 96-well plates and scanned using a confocal quantitative image cytometer (Yokogawa, Tokyo, Japan).

### Xenografts

We used 10 nude mice supplied by the Animal Center of Xi`an Jiaotong University. We injected 1 × 10^6^ oxaliplatin-resistant SGC-7901 cells, 5 transfected with NC lentivirus and 5 transfected with LINC00963 knockdown lentivirus, into the right flank of mice to generate xenografts. Four weeks later, xenograft tumours were harvested for further analysis. Our study was carried out in accordance with ARRIVE guidelines.

### Statistical analyses

All statistical analyses were performed using GraphPad Prism 8.2. Specifically, we employed Student’s *t*-test to analyse differences between two groups and one-way analysis of variance (ANOVA) for comparisons among multiple groups. Data with p < 0.05 were considered statistically significant.

## Results

### RNA sequencing was used to identify LINC00963 and miR-4458 as key regulators of local recurrence

**Figure 1:**
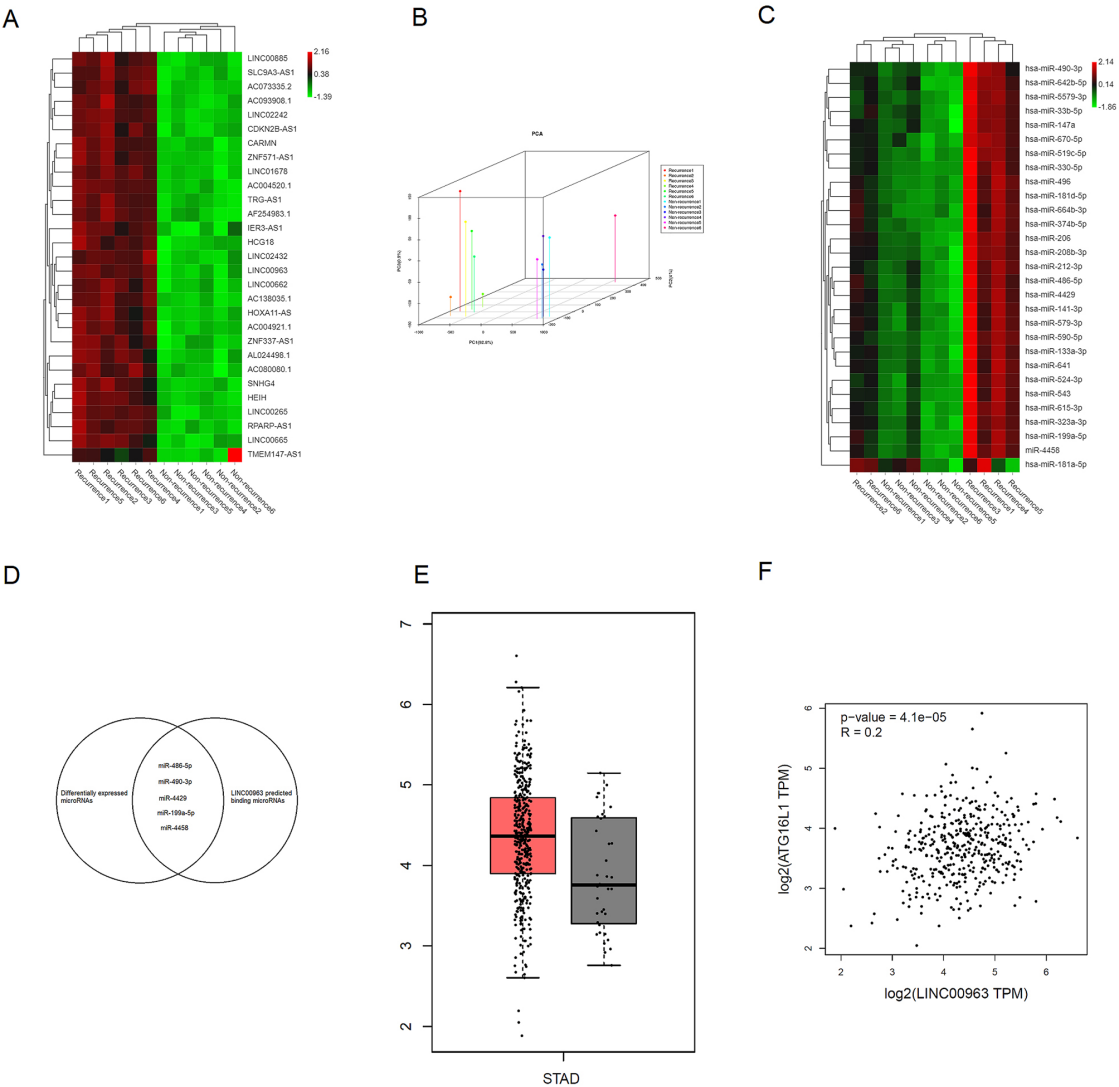
(**A**) Heatmap of the top 30 differentially expressed lncRNAs in gastric cancer patients with and without local recurrence. (**B**) Three-dimensional PCA of differentially expressed lncRNAs between gastric cancer patients with and without local recurrence. (**C**) Heatmap of the top 30 differentially expressed microRNAs between gastric cancer patients with and without local recurrence. (**D**) Venn plots to identify microRNAs that were involved in both differentially expressed microRNAs and predicted microRNAs. (**E**) LINC00963 expression in gastric cancer patients based on the GEPIA database. (**F**) The correlation between LINC00963 expression and ATG16L1 expression based on the GEPIA database.

RNA sequencing was used to evaluate the differentially expressed lncRNAs and microRNAs between gastric cancer patients with and without local recurrence (6 vs. 6). A heat map was plotted to exhibit the top 30 differentially expressed lncRNAs (Fig. 1A); the following 3D PCA plot is shown in Fig. 1B: LINC00963 contributed the most to PC1, which accounted for 92.8% of the between-group difference (Fig. 1B). The top 30 differentially expressed microRNAs are shown in Fig. 1C. A Venn plot was used to identify overlapping microRNAs between predicted microRNAs and differentially expressed microRNAs (Fig. 1D). Based on current evidence, autophagy is a major mechanism in regulating local recurrence. According to the GEPIA database, we found that LINC00963 was aberrantly highly expressed in gastric cancer tissues (Fig. 1E) and that LINC00963 expression positively correlated with ATG16L1 expression (Fig. 1F). Furthermore, we found that only miR-4458 and miR-4429 interacted with both LINC00963 and ATG16L1.

### Profiles of LINC00963/miR-4458 expression in gastric cancer patients and cell lines

**Figure 2:**
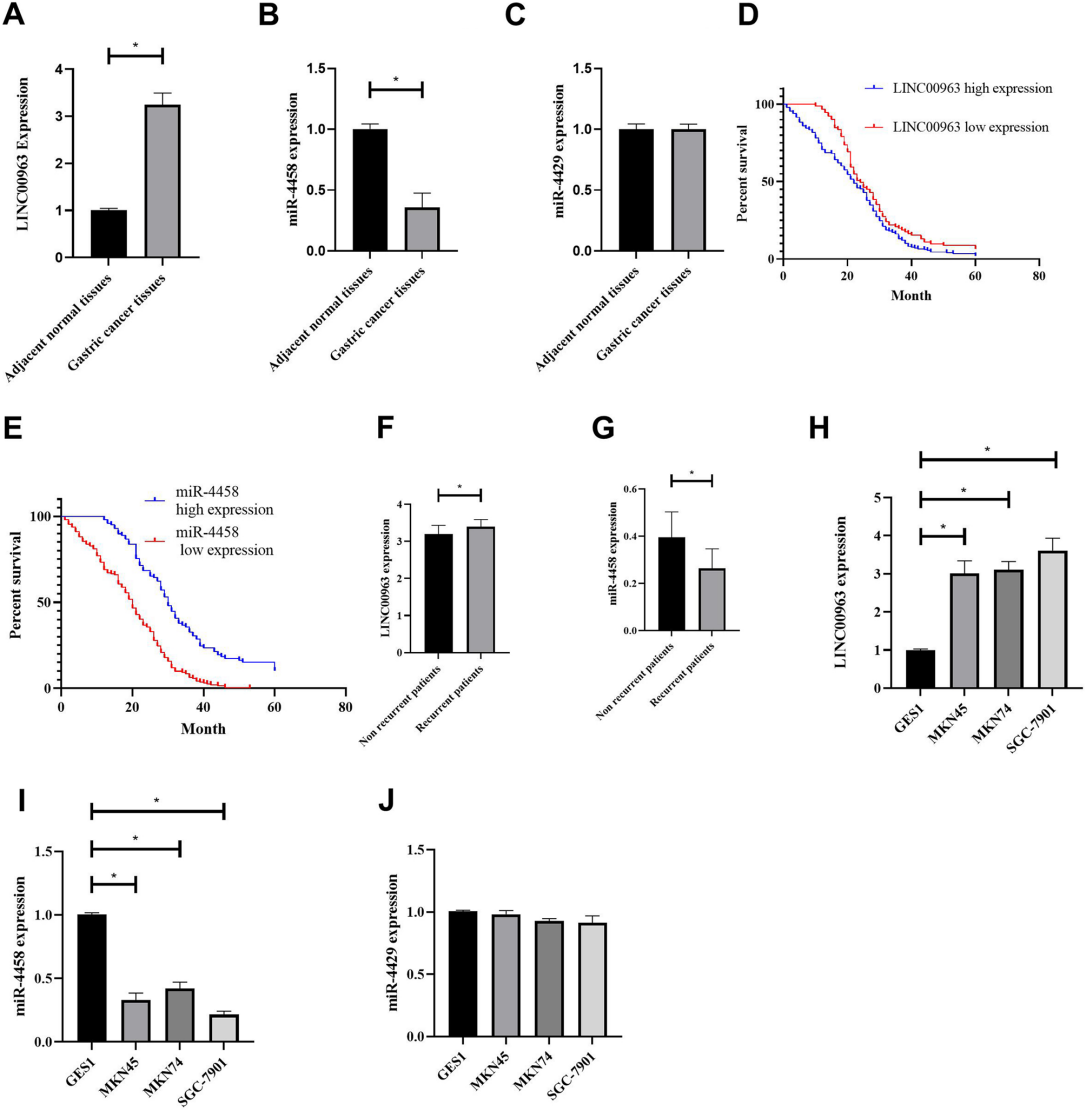
(**A**–**C**) LINC00963, miR-4458 and miR-4429 expression profiles in gastric cancer tissues compared with adjacent normal tissues. (**D**) and (**E**) Survival analysis for LINC00963 and miR-4458 expression in gastric cancer patients. (**F**) and (**G**) LINC00963 and miR-4458 expression in tissues from gastric cancer patients with and without local recurrence. (**H**–**J**) LINC00963, miR-4458 and miR-4429 expression in gastric cancer cells.

LINC00963 expression was higher and miR-4458 expression was lower in gastric cancer tissues than in adjacent normal tissues. (Fig. 2A and B). However, miR-4429 was not differentially expressed in gastric cancer tissues and normal tissues (Fig. 2C). Survival analysis revealed that high LINC00963 expression in patients or low miR-4458 expression in patients correlated with poor prognosis (Fig. 2D and E). Moreover, higher LINC00963 and lower miR-4458 levels were recorded in tissues from gastric cancer patients with local recurrence (Fig. 2F and G). In vitro experiments revealed higher LINC002381 expression in the SGC-7901, MKN45 and MKN74 cell lines than the GSE-1 cell line (Fig. 2H), as well as low miR-4458 levels in gastric cancer cells (Fig. 2I). However, miR-4429 was not significantly expressed in gastric cancer cells (Fig. 2J).

### LINC00963 knockdown sensitizes gastric cells to oxaliplatin treatment

**Figure 3:**
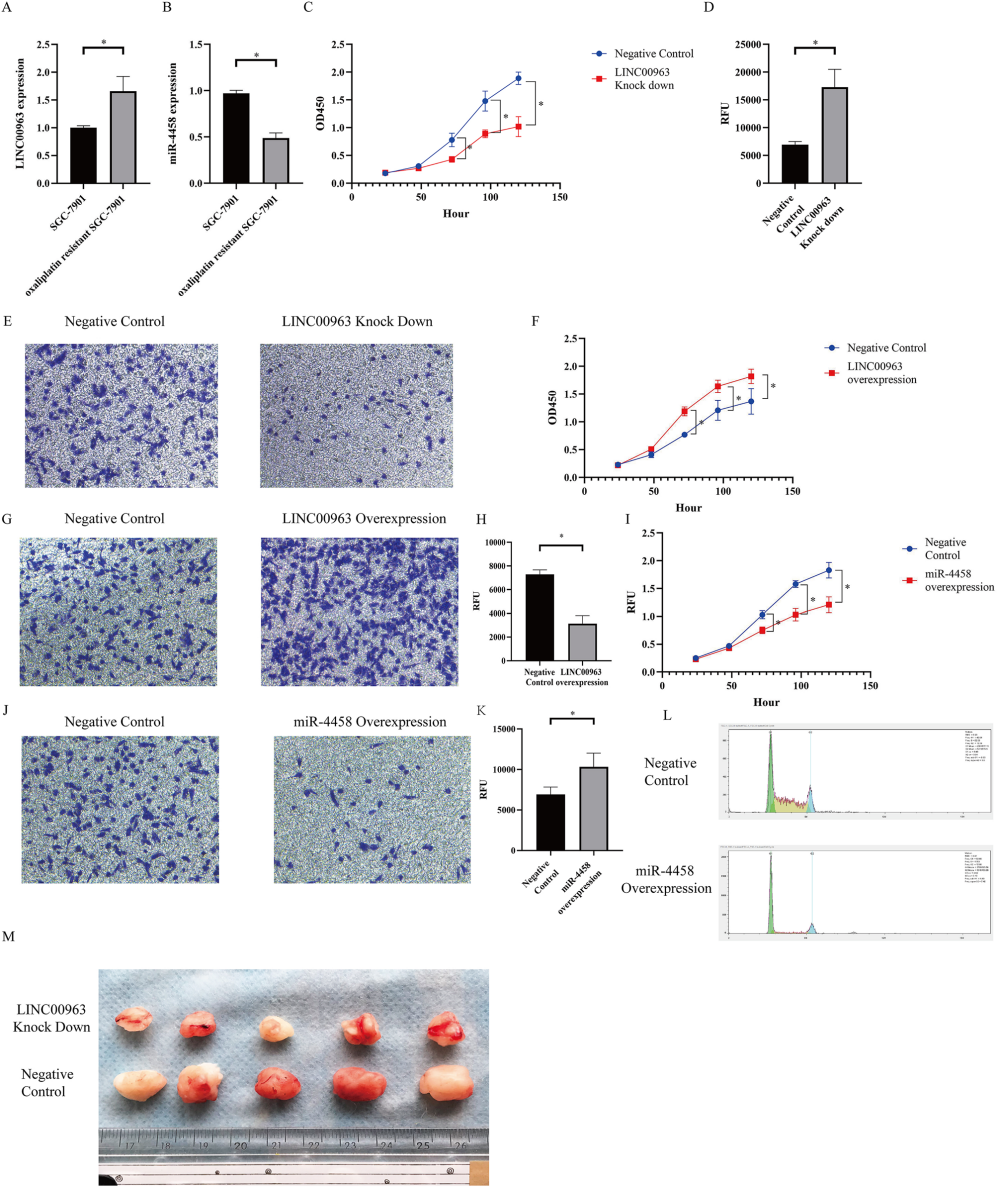
(**A**) LINC00963 expression in oxaliplatin-resistant SGC-7901 cells compared with SGC-7901 cells. (**B**) miR-4458 expression in oxaliplatin-resistant SGC-7901 cells compared with SGC-7901 cells. (**C**) Cell viability evaluated by CCK-8 assays of LINC00963 knockdown SGC-7901-R cells and negative control SGC-7901-R cells (transfected with control plasmids). (**D**) Cell apoptosis rate evaluated by Caspase 3/7 activity assays of LINC00963 knockdown and negative control SGC-7901-R cells (transfected with control plasmids). (**E**) Cell migration evaluated by Transwell assays of LINC00963 knockdown and negative control SGC-7901-R cells (transfected with control plasmids). (**F**) Cell viability evaluated by CCK-8 assays of LINC00963-overexpressing and negative control SGC-7901-R cells (transfected with control plasmids). (**G**) Cell migration evaluated by Transwell assays of LINC00963 overexpression and negative control SGC-7901-R cells (transfected with control plasmids). (**H**) Cell apoptosis rate evaluated by Caspase 3/7 activity assays of LINC00963 overexpression and negative control SGC-7901-R cells (transfected with control plasmids). (**I**) Cell viability evaluated by CCK-8 assays of miR-4458 overexpression and negative control SGC-7901-R cells (transfected with control plasmids). (**J**) Cell migration evaluated by Transwell assays of miR-4458 overexpression and negative control SGC-7901-R cells (transfected with control plasmids). (**K**) Cell apoptosis rate evaluated by Caspase 3/7 activity assays of miR-4458 overexpression and negative control SGC-7901-R cells (transfected with control plasmids). (**L**) Cell cycle detected by flow cytometric analysis of miR-4458-overexpressing and negative control SGC-7901-R cells (transfected with control plasmids). (**M**) In vivo experiments to show the effect of linc00963 knockdown on tumour growth.

LINC00963 expression was higher and miR-4458 expression was lower in the oxaliplatin-resistant SGC-7901 (SGC-7901-R) cell line (Fig. 3A and B). LINC00963 knockdown suppressed cell viability and promoted apoptosis in SGC-7901-R cells (Fig. 3C and D). In addition, impaired migration was detected in the LINC00963 knockdown SGC-7901-R cell line (Fig. 3E). Conversely, LINC00963 overexpression promoted the viability and migration of SGC-7901-R cells (Fig. 3F and G). In addition, LINC00963 overexpression suppressed oxaliplatin-induced apoptosis (Fig. 3H), whereas miR-4458 overexpression resulted in reduced cell proliferation and migration and enhanced apoptosis (Fig. 3I–K) of SGC-7901-R cells. Furthermore, miR-4458 overexpression led to G1 arrest (Fig. 3L).

In vivo experiments showed that LINC00963 knockdown could reduce the tumour growth of SGC-7901-R xenografts (Fig. 3M).

### miR-4458 is a downstream target for LINC00963

**Figure 4:**
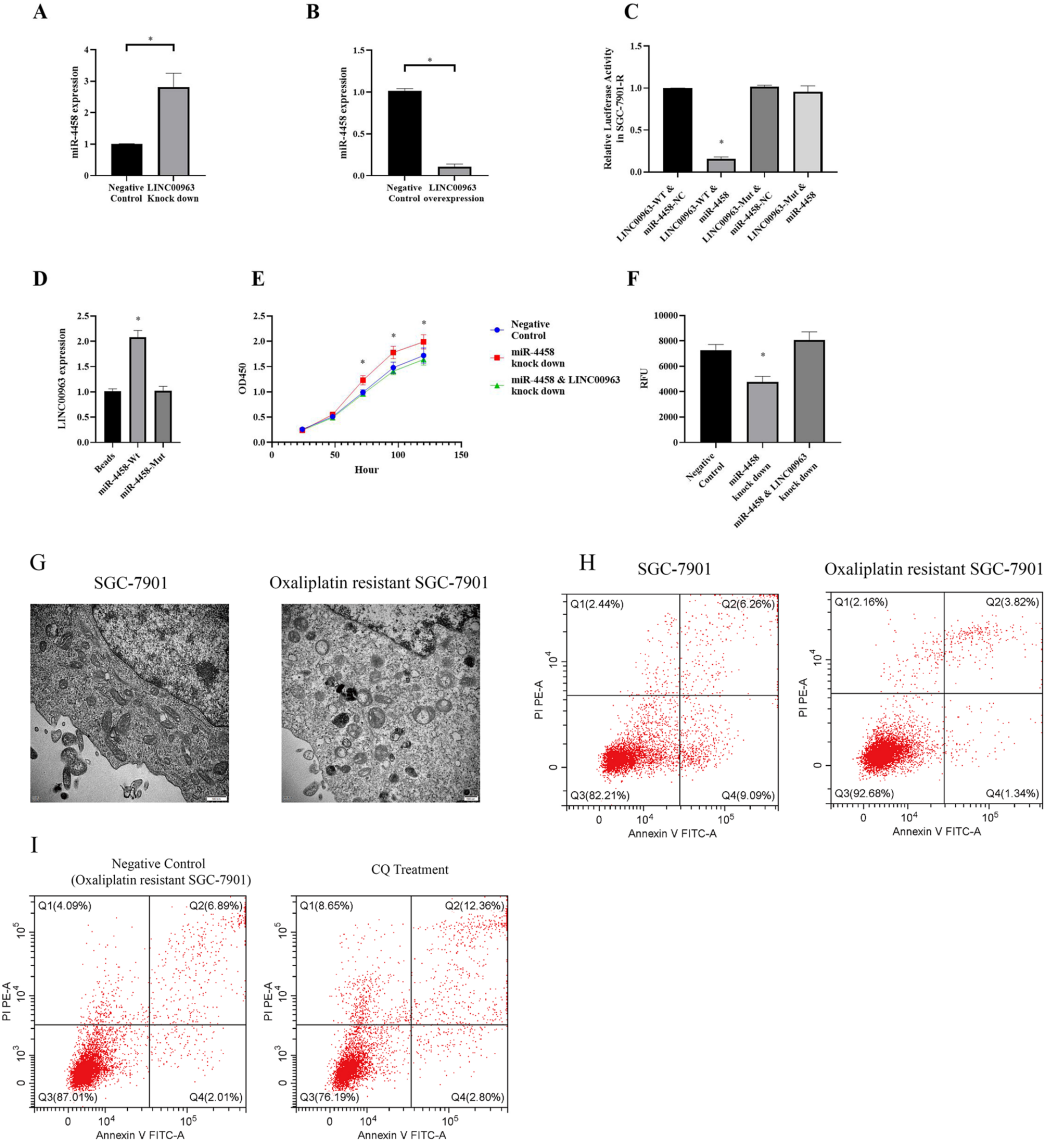
(**A**) miR-4458 expression in the LINC00963 knockdown SGC-7901-R cell line. (**B**) miR-4458 expression in the LINC00963-overexpressing SGC-7901-R cell line. (**C**) Dual luciferase reporter assays for miR-4458 and LINC00963 in SGC-7901-R cells. (**D**) RNA pulldown assays for miR-4458 and LINC00963 in SGC-7901-R cells. (**E**) Cell viability tested by CCK-8 assays of miR-4458 knockdown, miR-4458 and LINC00963 knockdown and negative control SGC-7901-R cells. Cells were transfected with control plasmids, miR-4458 knockdown plasmids, and miR-4458 knockdown plasmids and LINC00963 knockdown plasmids. (**F**) Cell apoptosis tested by caspase-3/7 analysis of miR-4458 knockdown, miR-4458 and LINC00963 knockdown and negative control SGC-7901-R cells. Cells were transfected with control plasmids, miR-4458 knockdown plasmids, and miR-4458 knockdown plasmids and LINC00963 knockdown plasmids. (**G**) Transmission electron microscopy to evaluate autophagosomes in SGC-7901 and SGC-7901-R cells. (**H**) Flow cytometry to evaluate cell apoptosis in SGC-7901 and SGC-7901-R cells. I Flow cytometry to evaluate cell apoptosis in SGC-7901-R cells treated with or without chloroquine.

LINC00963 knockdown induced a significant increase in miR-4458 expression in SGC-7901-R cells (Fig. 4A), whereas upregulated LINC00963 expression led to downregulation of miR-4458 expression (Fig. 4B). The results from the dual luciferase reporter assay indicated that LINC00963 directly interacted with miR-4458 in oxaliplatin-resistant SGC-7901 cells (Fig. 4C). Furthermore, an RNA pulldown assay showed that LINC00963 bound to miR-4458 and regulated its expression (Fig. 4D) in oxaliplatin-resistant SGC-7901 cells, whereas rescue experiments showed that downregulating miR-4458 expression could reverse the LINC00963-mediated cell proliferation and apoptosis (Fig. 4E,F) of oxaliplatin-resistant SGC-7901 cells.

As shown in Fig. 4G, we found enhanced autophagy in SGC-7901-R cells compared with SGC-7901 cells by transmission electron microscopy (Fig. 4G), indicating that autophagy might play an important role in regulating oxaliplatin resistance. In addition, we found a reduced apoptosis rate in SGC-7901-R cells compared with SGC-7901 cells (Fig. 4H). Furthermore, after treatment with chloroquine (CQ), an inhibitor of autophagy, we found that the apoptosis rate was induced in SGC-7901-R cells, indicating that an inhibitor of autophagy might reverse oxaliplatin resistance by promoting oxaliplatin-induced apoptosis (Fig. 4I).

### LINC00963 knockdown suppressed autophagy by downregulating ATG16L1 expression

**Figure 5:**
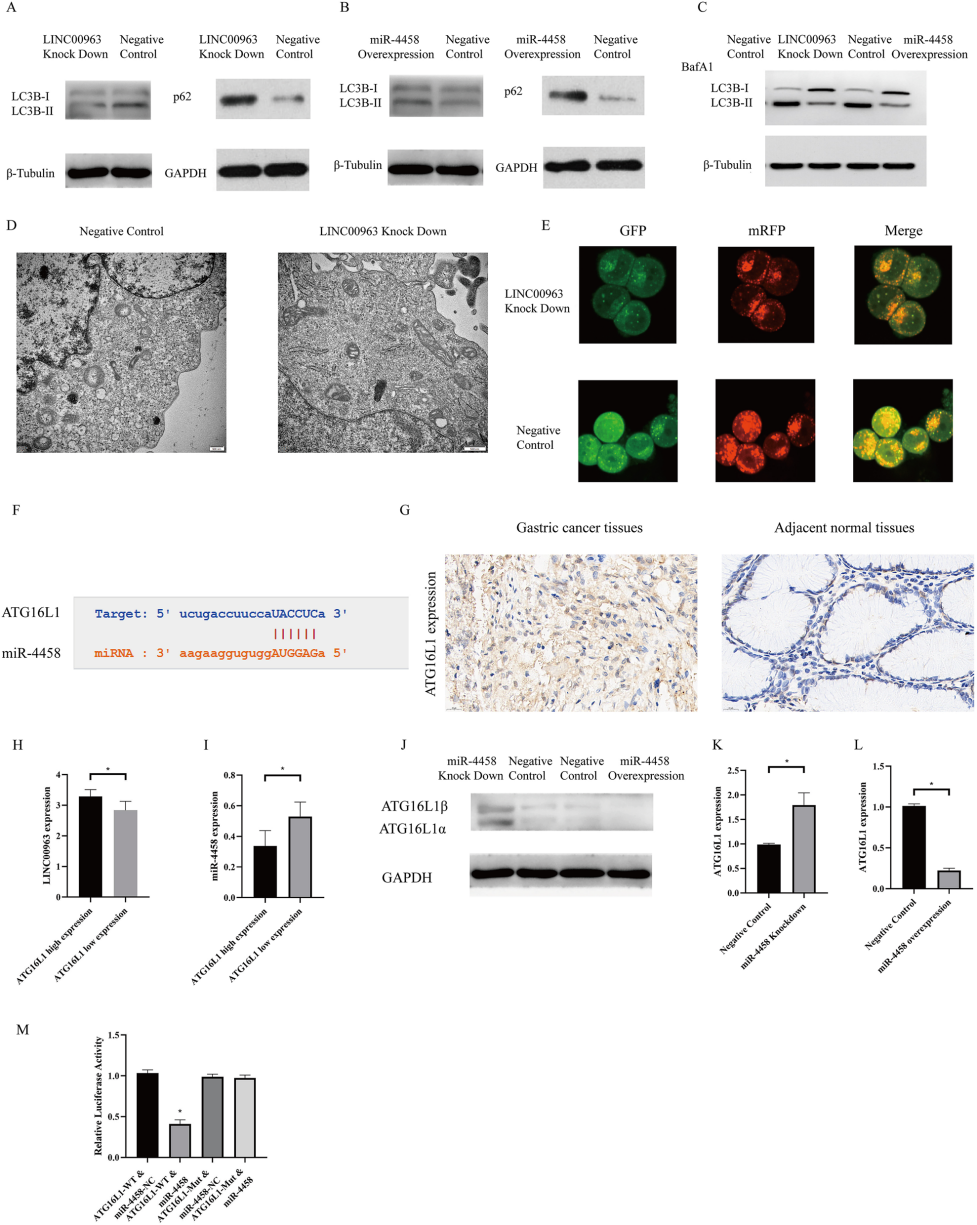
(**A**) and (**B**) Western blots to test the expression of LC3 and p62 in the LINC00693 knockdown and miR-4458 overexpression SGC-7901-R cell lines. (**C**) Western blots to test the expression of LC3 in LINC00693 knockdown and miR-4458 overexpression SGC-7901-R cells treated with BafA1. (**D**) Transmission electron microscopy to evaluate autophagosomes in SGC-7901-R (negative control) and LINC00963 knockdown SGC-7901-R cells. (**E**) Dual fluorescent lentivirus autophagy system to detect autophagic flux in SGC-7901-R (negative control) and LINC00963 knockdown SGC-7901-R cells. (**F**) Predicted binding sites of miR-4458 to ATG16L1 by starBase V3.0. (**G**) Expression of ATG16L1 in gastric cancer patients. (**H**) Linc00963 expression between gastric cancer patients with high ATGL1 expression and those with low ATGL1 expression. I miR-4458 expression between gastric cancer patients with high ATGL1 expression and those with low ATGL1 expression. (**J**) Expression of ATG16L1 in SGC-7901-R cells by Western blot after knocking down or overexpressing miR-4458. (**K**) Expression of ATG16L1 in SGC-7901-R cells by qRT-PCR after knocking down miR-4458. (**L**) Expression of ATG16L1 in SGC-7901-R cells by qRT-PCR after overexpressing miR-4458. (**M**) Dual luciferase reporter assays for miR-4458 and ATG16L1 in SGC-7901-R cells.

LINC00963 knockdown resulted in a significant decrease in LC3II expression and an increase in p62 levels, indicating suppression of autophagy (Fig. 5A), whereas overexpressing miR-4458 suppressed autophagic flux (Fig. 5B). Moreover, after treatment with bafilomycin, we found accumulated LC3B-I in LINC00963 knockdown or miR-4458 overexpression SGC-7901-R cells, indicating activated autophagic flux in SGC-7901-R cells (Fig. 5C). Furthermore, transmission electron microscopy (Fig. 5D) and a dual fluorescent lentivirus autophagy system (Fig. 5E) were used to show that LINC00963 knockdown could suppress the autophagic flux of SGC-7901-R cells. Among all starBase V3.0-predicted targets of miR-4458, miR-4458 might bind to the 3' UTR of the autophagy-related gene ATG16L1 (Fig. 5F). ATG16L1 was also overexpressed in gastric cancer patients (Fig. 5G).

In addition, ATG16L1 expression was positively correlated with LINC00963 expression and negatively correlated with miR-4458 expression in vivo (Fig. 5H and I). Consequently, downregulating miR-4458 expression resulted in ATG16L1 overexpression at both the protein and mRNA levels (Fig. 5J and K). In addition, upregulating miR-4458 expression resulted in a decrease in ATG16L1 expression (Fig. 5J and L), whereas a dual luciferase reporter assay revealed that miR-4458 directly interacted with ATG16L1 (Fig. 5M).

## Discussion

To date, several long noncoding RNAs that regulate the initiation and progression of cancers have been identified^17^. Gastric cancer is commonly known for its tendency to recur and is mainly controlled by platinum, especially oxaliplatin, treatment^2^. In the present study, we evaluated the role of LINC00963 as a novel oncogene and analysed its potential in reversing oxaliplatin resistance. We found aberrantly high expression of LINC00963 in gastric cancer patients with recurrence, which led to poor prognosis due to local recurrence. Based on current knowledge, we hypothesized that LINC00963 might promote local recurrence of gastric cancer by inducing oxaliplatin resistance. Generally, oxaliplatin exerts its anticancer functions by inducing DNA damage and cell apoptosis^3^. Under this circumstance, Zhang et al. demonstrated that LINC00963 knockdown induced DNA damage and oxidative stress, thereby improving the radiation sensitivity of breast cancer cells^22^. Based on this, LINC00963 might induce oxaliplatin resistance. In this study, we found that LINC00693 knockdown could improve the oxaliplatin sensitivity of gastric cancer cells and was also associated with the activation of autophagic flux by interacting with miR-4458.

Previous studies have shown that activation of autophagic flux can either promote or inhibit oxaliplatin resistance^12,23^. For instance, Jeong et al.^23^ found that NOS3- and SOD2-induced autophagy was essential for the development of oxaliplatin resistance in colorectal cancer cells. Moreover, they reported that cannabidiol, an autophagic flux enhancer, significantly sensitized cancer cells to oxaliplatin. However, numerous studies have identified inhibition of autophagy in enhanced drug sensitivity^24^. In this study, we found enhanced autophagy in oxaliplatin-resistant gastric cancer cells, indicating that activated autophagy might be important in inducing oxaliplatin resistance. Oxaliplatin mainly suppresses cancer progression by inducing apoptosis, and we found decreased apoptosis in oxaliplatin-resistant cells, indicating that autophagy might be associated with oxaliplatin-induced apoptosis. More importantly, autophagic cell death, including autophagy-related apoptosis, assists cells in resisting potential hazards, such as platinum treatment, and finally results in drug resistance^25^. In this respect, targeting autophagy, such as with chloroquine, has been considered a feasible way to reverse chemotherapy resistance^6^. Our study further utilized chloroquine to suppress autophagy in SGC-7901-R cells and found a significant increase in the apoptosis rate; this phenomenon might clarify how autophagy inhibitors reverse oxaliplatin resistance. However, clarifying the interaction of autophagic cell death and oxaliplatin-induced apoptosis is complicated. In this study, it was evident that autophagic inhibition in gastric cancer improved oxaliplatin sensitivity, whereas LINC00963 knockdown induced downregulation of ATG16L1 expression, thereby improving the effects of oxaliplatin. Overall, these findings indicate that targeting autophagy to aid in oxaliplatin treatment during clinical practice is complex and requires more studies to elucidate the underlying mechanisms.

Prediction of potential LINC00693 and ATG16L1 targets using starBase V3.0 revealed that miR-4458 could interact with both genes^26^. However, only a handful of studies have implicated miR-4458 in multidrug resistance. Our results indicated that miR-4458 was associated with local recurrence of gastric cancer in vivo and enhanced oxaliplatin’s anticancer effects in vitro*,* in line with previous studies. For example, Ma et al.^27^ demonstrated that miR-4458 inhibits the migration of non-small-cell lung cancer cells by suppressing epithelial-mesenchymal transition through downregulation of HMGA1 expression. Similarly, HMGA1 was reported to be an important negative autophagic regulator owing to its ability to suppress autophagosome formation^28^. Our results showed that miR-4458 directly interacted with ATG16L1, thereby impairing autophagosome formation. However, more studies are needed to elucidate the mechanism underlying the miR-4458 and ATG16L1 interaction, which might provide novel therapeutic targets.

In conclusion, our results indicated that LINC00963 and miR-4458 are important indicators for the overall survival and local recurrence of gastric cancer patients. Functionally, the results from in vitro experiments demonstrated that LINC00963 binds to and negatively regulates miR-4458, which subsequently interacts with ATG16L1’s 3' UTR to enhance oxaliplatin resistance by activating autophagy.

## Supplementary Information

Supplementary Information.
