# Supplementary material for: LINC00963/miR-4458 regulates the effect of oxaliplatin in gastric cancer by mediating autophagic flux through targeting of ATG16L1
Source: Sci Rep. 2021 Oct 25;11:20951. doi: 10.1038/s41598-021-98728-9 (PMC8546147; doi:10.1038/s41598-021-98728-9)

Western blot for LC3 in Figure 5A and 5B


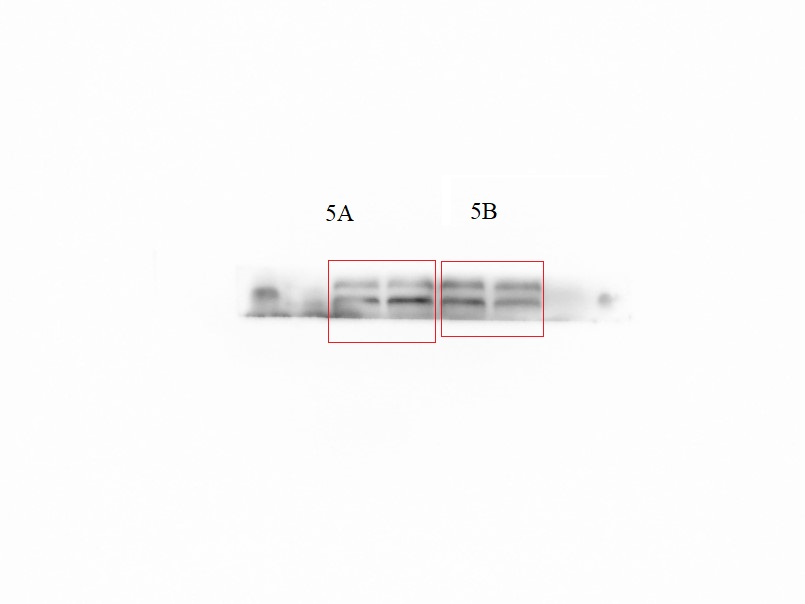


Western blot for β-tubulin in Figure 5A and 5B


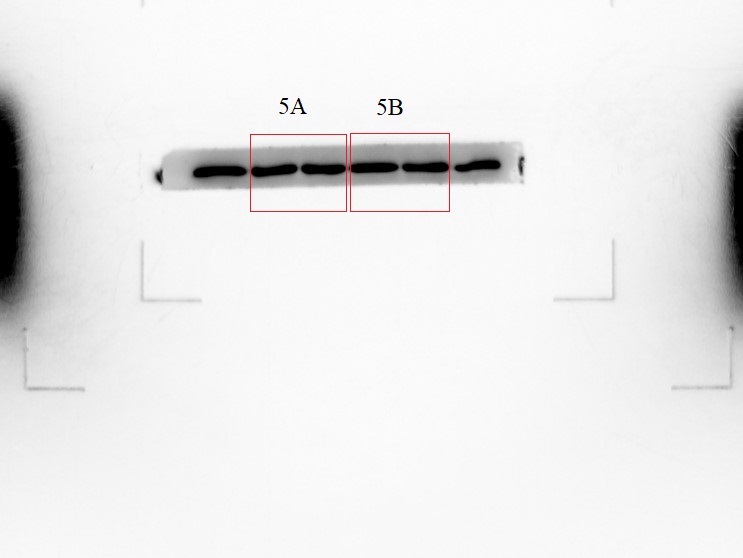


Western blot for GAPDH in Figure 5A and 5B (The last membrane)

Western blot for p62 in Figure 5A and 5B （The third membrane）


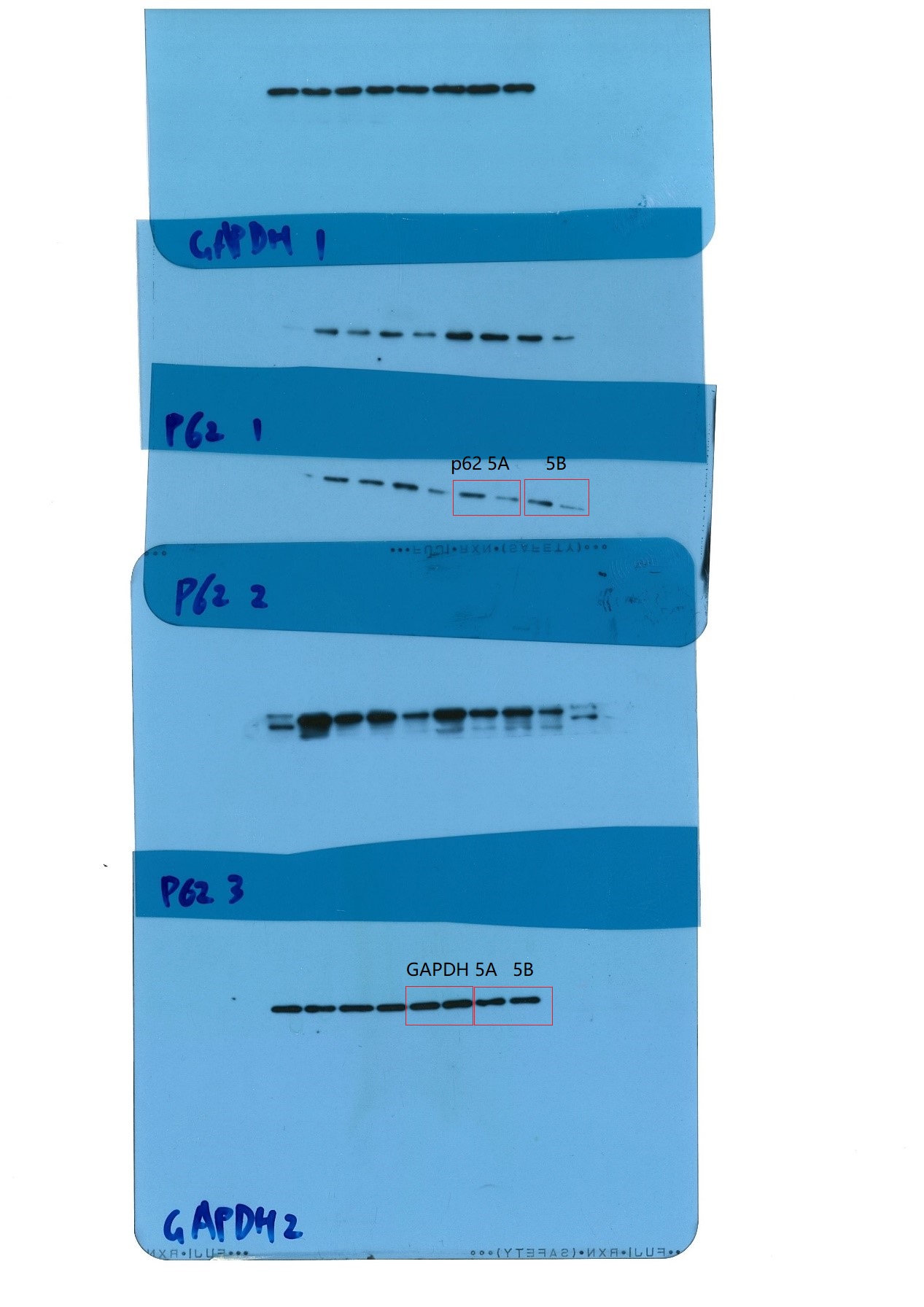


Western blot for LC3 in Figure 5C


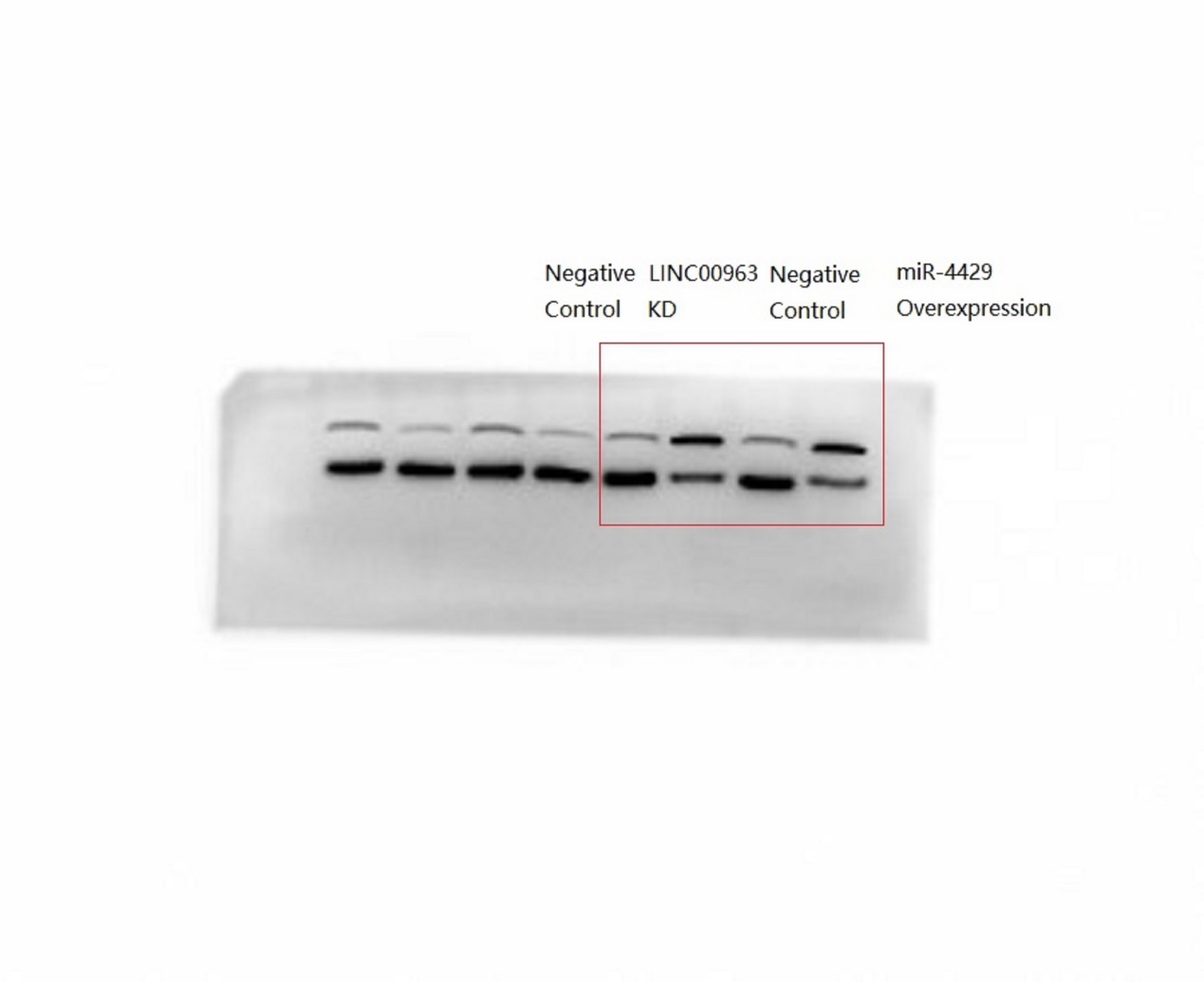


Western blot for β-tubulin in Figure 5C


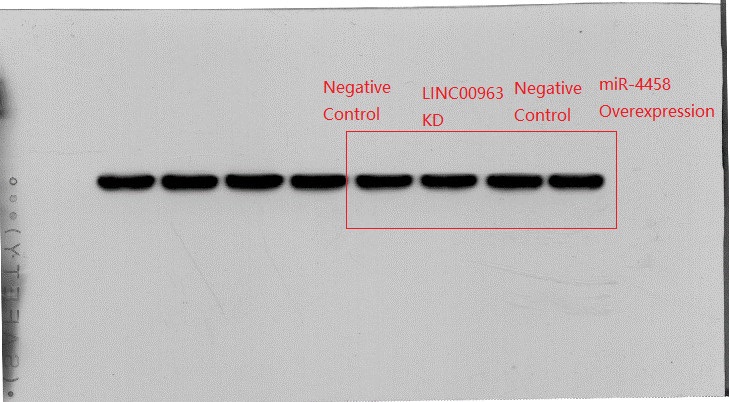


Western blot for ATG16L1 in Figure 5J


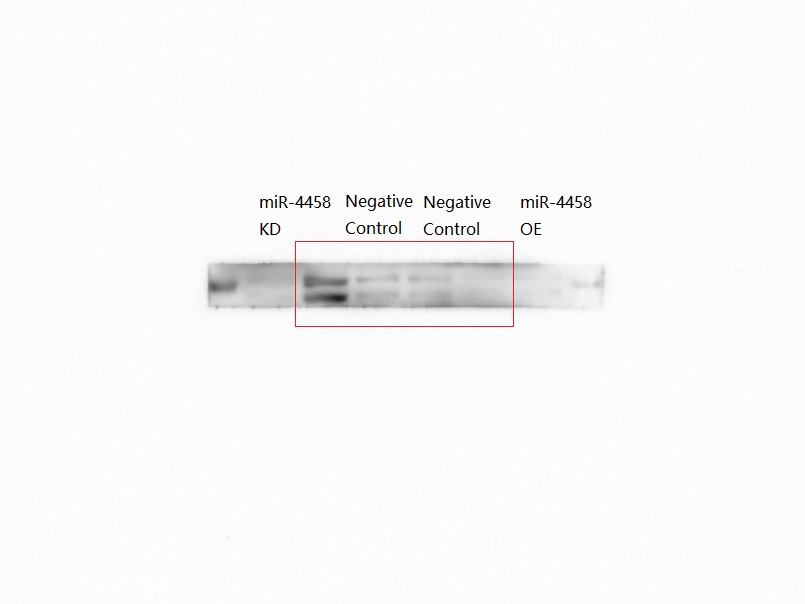


Western blot for GAPDH in Figure 5J


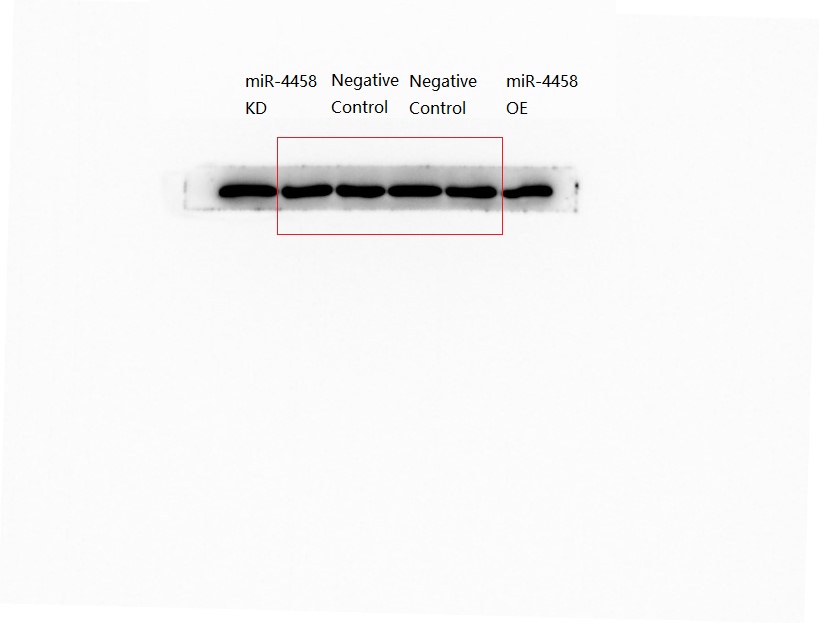

Supplement: Supplementary file 1 — Supplementary Information. [file 41598_2021_98728_MOESM1_ESM.docx]
